# Blockchain enabled deep learning model with modified coati optimization for sustainable healthcare disease detection and classification

**DOI:** 10.1038/s41598-025-06578-6

**Published:** 2025-07-01

**Authors:** Heba G. Mohamed, Fadwa Alrowais, Fahd N. Al-Wesabi, Mesfer Al Duhayyim, Anwer Mustafa Hilal, Abdelwahed Motwakel

**Affiliations:** 1 Department of Electrical Engineering, College of Engineering, Princess Nourah bint Abdulrahman University, P.O. Box 84428, 11671 Riyadh, Saudi Arabia; 2 Electrical Department, College of Engineering, Alexandria Higher Institute of Engineering and Technology, Alexandria, 21421 Egypt; 3 Department of Computer Sciences, College of Computer and Information Sciences, Princess Nourah bint Abdulrahman University, P.O. Box 84428, 11671 Riyadh, Saudi Arabia; 4 Department of Computer Science, Applied College at Mahayil, King Khalid University, Mahayil Aseer, Saudi Arabia; 5 Department of Computer Science, College of Computer Engineering and Sciences, Prince Sattam Bin Abdulaziz University, 16273 Al-Kharj, Saudi Arabia; 6 Department of Computer and Self Development, Preparatory Year Deanship, Prince Sattam Bin Abdulaziz University, AlKharj, Saudi Arabia; 7 Department of Management Information Systems, College of business administration in Hawtat bani Tamim, Prince Sattam Bin Abdulaziz University, Al-Kharj, Saudi Arabia

**Keywords:** Diseases, Engineering, Mathematics and computing

## Abstract

The growing number of patients and the emergence of new symptoms and diseases make health monitoring and assessment increasingly complex for medical staff and hospitals. The execution of big and heterogeneous data gathered by medical sensors and the necessity of patient classification and disease analysis have become serious problems for various health-based sensing applications. The significant features of healthcare are the privacy of medical details and the accuracy of disease identification. One of the key benefits of the healthcare system is the ability to predict diseases early. Recently, the progress of artificial intelligence (AI) in the healthcare system has been a high priority. Machine learning (ML) and deep learning (DL) effectively make analyses and strategic decisions for the healthcare system. This manuscript proposes a Modified Coati Optimization Driven Blockchain for Healthcare Disease Detection and Classification (MCODBC-HDDC) method. The presented MCOBC-HDDC method provides an efficient and accurate disease diagnosis, utilizing a system that depends on DL techniques. Initially, the MCODBC-HDDC method incorporates BC technology to ensure secure data sharing and management, providing a decentralized and tamper-proof environment for patient data. In the data preprocessing stage, the MCODBC-HDDC model employs Z-score normalization to standardize the data and improve performance. For the optimal subset of features, the spotted hyena optimization algorithm (SHOA) model is used. Furthermore, the attention bidirectional gated recurrent unit (ABiGRU) method is implemented for disease detection and classification. Finally, the hyperparameter selection of the ABiGRU method is performed by utilizing the modified coati optimization algorithm (MCOA) method. The experimental analysis of the MCODBC-HDDC approach is examined under the HD dataset. The performance validation of the MCODBC-HDDC approach portrayed a superior accuracy value of 97.36% over existing models.

## Introduction

The healthcare industry creates various data about patients, diagnoses, and diseases, but it is not properly examined, so it does not present the value that needs to be. Heart disease is the main cause of death^1^. According to the WHO, cardiovascular disease (CVD) is the important reason of death globally, causing the fatalities of approximately 17.9 million persons yearly. The healthcare sector creates various data about patients, diagnoses, and diseases, but it is not appropriately analyzed, so it does not have a similar effect as it should on patient well-being^2^. Additionally, socioeconomic factors like money and jobs have a great impact on the mortality rate by the effect on contraindications associated with lifestyle after and before cardiac illness. The greatest way to reduce such deaths is to identify them instantly^3^. The capability to predict the occurrence of heart illness can be crucial for managing the required treatment without delay. The decentralized process of providing security and incorporation is resolved over Blockchain (BC). The BC offers probable advantages by encrypting the data kept, and also every block can be e-signed to deliver a higher level of accuracy^4^. BC is an appropriate solution for the healthcare sector as it contains various performers and needs a great level of trust among the performers. The Healthcare industry is an important domain in the present information and communication technology domain. Nowadays, remote patient monitoring and digital health records are probable by the IoT in the healthcare sector^5^. The healthcare data created by different resources were widespread and are in various methods that increase anguishes regarding the data quality. Also, it has the potential to utilize health data in various applications, like prediction of disease^6^. Therefore, data quality must be assured while incorporating the data from numerous devices, and it is quite challenging. The problem of data privacy occurs once the healthcare data are distributed across the network, and single-point failure is probable when the data are kept in a leading centralized position. BC is a distributed storage, whose goal is to synchronize data across healthcare sources^7^.

Alternatively, Deep learning (DL) is a subcategory of artificial intelligence (AI), which utilizes intricate neural networks (NNs) for extracting insights and patterns from massive quantities of data^8^. The main benefit of utilizing DL techniques in combination with BC is the capability to analyze and process various kinds of data, with wearable sensor data, medical imaging, genomic information, and electronic health records^9^. DL methods could learn from these heterogeneous and rich data to recognize risk factors, biomarkers, and patterns related to different neurological illnesses like Parkinson’s disease, Alzheimer’s illness, epilepsy, and multiple sclerosis^10^. This interoperability enables the making of widespread datasets, which improve the precision and strength of DL methods, inducing enhanced diagnostic abilities.

The growing volume of medical data presents a valuable opportunity to improve disease detection and classification through intelligent analysis. Several data are improperly utilized due to restrictions in conventional processing methods and concerns over data security. Advanced technologies such as DL can extract crucial patterns for precise diagnosis, while BC ensures secure and transparent data management. Integrating these technologies presents a robust framework for enhancing clinical decision-making and patient care. This study is motivated by the requirement for a robust, secure, and efficient model to address the challenges of modern disease detection and classification in healthcare systems.

This manuscript proposes a Modified Coati Optimization Driven Blockchain for Healthcare Disease Detection and Classification (MCODBC-HDDC) method. The presented MCOBC-HDDC method provides an efficient and accurate disease diagnosis, utilizing a system that depends on DL techniques. Initially, the MCODBC-HDDC method incorporates BC technology to ensure secure data sharing and management, providing a decentralized and tamper-proof environment for patient data. In the data preprocessing stage, the MCODBC-HDDC model employs Z-score normalization to standardize the data and improve performance. For the optimal subset of features, the spotted hyena optimization algorithm (SHOA) model is used. Furthermore, the attention bidirectional gated recurrent unit (ABiGRU) method is implemented for disease detection and classification. Finally, the hyperparameter selection of the ABiGRU method is performed by utilizing the modified coati optimization algorithm (MCOA) method. The experimental analysis of the MCODBC-HDDC approach is examined under the HD dataset. The key contribution of the MCODBC-HDDC approach is listed below.

- The MCODBC-HDDC model integrates BC to ensure secure, decentralized, and tamper-proof sharing and management of patient data. It improves data integrity and trust across healthcare systems, facilitating reliable stakeholder communication while safeguarding sensitive medical information.
- The MCODBC-HDDC approach implements the Z-score normalization to improve data preprocessing by standardizing features to a standard scale. This improves model convergence and mitigates the influence of outliers. The model also confirms more reliable input for subsequent feature selection and classification stages.
- The MCODBC-HDDC method utilizes the SHOA technique for optimal feature selection by detecting the most relevant attributes in the dataset. This mitigates computational complexity and improves the model’s efficiency. Removing redundant or less informative features strengthens the overall predictive performance.
- The MCODBC-HDDC methodology implements the ABiGRU model to improve the accuracy of disease detection and classification. It captures contextual dependencies in both forward and backward sequences, improving the model’s capability to comprehend complex temporal patterns in patient data.
- The MCODBC-HDDC method employs the MCOA model to enable precise fine-tuning of model hyperparameters, resulting in improved predictive performance. It balances exploration and exploitation during optimization, resulting in a more robust and well-generalized disease prediction model.
- The novelty of the MCODBC-HDDC model is its integration of BC-based security, SHOA-driven feature selection, and MCOA-tuned ABiGRU classification. This integration creates a highly secure, efficient, and explainable framework for healthcare prediction. The model ensures robust performance and reliable data management by incorporating blockchain, optimization techniques, and advanced DL. This integration represents a significant advancement in secure and accurate healthcare forecasting.

## Related works

This section reviews recent advancements integrating BC and DL in healthcare data management and security. Several studies have proposed innovative models that enhance privacy, classification accuracy, and secure data sharing. For example, Ragab et al.^11^ propose a BC-driven privacy-preserving analysis of EHR utilizing a sine cosine algorithm (SCA) with a DL method called the BPEHR-SCADL model. The BPEHR-SCADL method mainly models an artificial fish swarm algorithm (AFSA) with a sign-cryption model to safely transfer EHRs. Furthermore, the BPEHR-SCADL model utilizes BC technology to store patients’ medical and official data in an exterior database. Also, the SCA with a deep feedforward neural network (DFFNN) technique can be used for the process of classification. Moreover, the SCA is used to optimally alter the bias and weight values of the DFFNN method. Ali et al.^12^ introduce a permission-based BC structure for extensible and secure healthcare systems, incorporating hybrid DL methods. The structure guarantees that only approved individuals can modify and access sensitive health data, maintaining patient confidentiality while enabling continuous data sharing and association among healthcare sources. Alanazi et al.^13^ suggest a new BC with an optimum DL-based safe data sharing and classification (BCODL-SDSC) model in the upcoming healthcare system. Initially, the BCODL-SDSC model allows BC technology to store and preserve patient information from the process of numerous communications and facilitate access control to the numerous participants.

Alamro et al.^14^ introduce BC Aided IoT Healthcare System utilizing Ant Lion Optimizer with Hybrid DL (BHS-ALOHDL) method. The BHS-ALOHDL approach implements the ALO ALO-based feature subset selection (ALO-FSS) model to create sequences of feature vectors. Mohananthini et al.^15^ propose a novel BC-aided heart illness recognition and classification method with feature selection (FS) with optimum fuzzy logic (BHDDC-FSOFL) system. The suggested BHDDC-FSOFL model utilizes BC technology to store healthcare information safely. Furthermore, the illness recognition module contains the model of the learning-based optimizer (BTLBO) method and biogeography teaching for the FS process. In addition, the ebola search optimizer (ESO) method can be utilized for the ANFIS classifier parameter tuning.

Qu et al.^16^ present a quantum arrhythmia detection system named QADS. Additionally, a quantum NN was utilized in QADS to identify unusual ECG data that is given to advance CVD diagnosis. Every quantum block saves the present and preceding block hash to create a quantum block network. The novel quantum BC method presents a managed quantum walk hash function and a quantum verification protocol for assuring security and validity in making novel blocks. Also, this study creates a hybrid quantum CNN named HQCNN for extracting the temporal features of ECG to identify unusual heart rates. Naveen et al.^17^ present an innovative solution by incorporating the DenseNet structure with BC technology to improve the recognition of melanoma. Using a quantitative method, the research uses the DenseNet method that features dense connectivity transition layers, and bottleneck layers to enhance feature representation while reducing computational difficulty and size of the model. The method is additionally improved by transfer learning (TL), pre-trained on a dermoscopic image dataset.

Kateb et al.^18^ presented the enhanced security mechanism for human-centred systems using deep learning with a jellyfish search optimizer (ESHCS-DLJSO) approach for secure IoT healthcare. The model employs min–max normalization, bacterial foraging optimization algorithm (BFOA) for feature extraction, a convolutional neural network with long short-term memory and attention (CNN-LSTM-Attention) for disease detection, and jellyfish search optimizer (JSO) for hyperparameter tuning. Rahal et al.^19^ proposed a BC-based approach for securely sharing locally trained DL methods across healthcare sectors. Hota et al.^20^ proposed a customized CNN-based intelligent diagnosis system. The model comprises hospital registration via smart contracts, disease diagnosis, and secure learning parameters, ensuring data privacy and attack resilience. Rokade and Mishra^21^ proposed a BC-based system for classifying skin diseases using an optimized DL method, integrating a novel transit circle-inspired optimization (TCIO) model for LeNet and a modified DeepJoint segmentation technique. Islam et al.^22^ explored the utilization of ML and AI in early disease detection, healthcare optimization, and predictive analytics.

Shammi et al.^23^ reviewed the role of AI, ML, and BC in disease diagnosis and monitoring. It highlights the potential of these technologies to improve patient outcomes, enhance data security, and empower patients to control their health information. Matlo et al.^24^ introduced the proof of work validation with an explainable artificial intelligence (PoWV-XAI) approach, a BC-based validation scheme enhanced with XAI for healthcare applications. Khan and Jilani^25^ presented a privacy-preserving heart disease prediction system that integrates cryptographic techniques, BC, and DL on a cloud platform. The system ensures secure data handling and achieves high prediction accuracy for heart disease. Data is decrypted and input into the heart disease prediction system (HDPS), which uses an artificial neural network (ANN), CNN, and RNN for preprocessing, feature extraction, and selection. Kulkarni et al.^26^ presented a BC-based IoT model utilizing the Aquila hunter-prey optimization (AHPO) method to generate a privacy matrix integrated with medical data. The model also implements data augmentation through oversampling and employs a Deep Maxout Network with SpinalNet (Spinal PMDMNN) model for healthcare classification.

Gupta et al.^27^ proposed a framework integrating BC technology and federated learning (FL) to enable secure, collaborative lung disease classification while preserving data privacy. The model attains high accuracy using the DenseNet-201 model and IPFS for storage. Ahmed et al.^28^ improved smart healthcare services using the PSO-BioBERT model for named entity recognition and relation extraction tasks from clinical records. Dubey, Kapoor, and Saraswat^29^ enhanced early disease diagnosis and risk reduction using hybrid learning methods, biological feature selection, BC data storage, EEG analysis, bidirectional LSTM for time-series data, and advanced medical image processing, including Alzheimer’s diagnosis and image segmentation. Chougule et al.^30^ incorporated BC-based approaches with feature selection techniques utilizing entropy and correlation coefficients to improve healthcare prediction models’ accuracy, efficacy, and security using cloud-stored E-Health data from the IoT. Al-Marridi, Mohamed, and Erbad^31^ optimized healthcare decision-making, enhanced security, reduced latency, and minimized costs using BC technology, deep reinforcement learning (DRL), multi-agent Q-Learning, and mixed decentralized Markov decision process for improved Quality of Service (QoS) and resource utilization. Pradhan et al.^32^ explored the utilization of ML models in healthcare systems, specifically in healthcare IoT (HIoT) and EHR data, which are the key data resources for NN models while addressing challenges such as security and service quality improvement. Table 1 summarizes the existing studies on NN models in healthcare disease detection and classification.

**Table 1:** Summary of existing studies integrating BC and DL models in healthcare applications.

Authors	Techniques	Metrics	Dataset	Major findings and limitations
Ragab et al.^11^	BC, SCA, DFFNN, AFSA, Signcryption Technique	Accuracy, Precision, Recall, F1-Score	Heart Statlog, Pima Indians Diabetes, and EEG Eye State	BPEHR-SCADL outperforms existing methods in secure and accurate EHR classification
Ali et al.^12^	BC, Hybrid DL, Permissions-based Framework	Scalability, Security, Data Interoperability, Diagnostic Accuracy, Privacy Preservation	Standard Dataset	The framework improves secure, scalable healthcare data management and accurate decision-making
Alanazi et al.^13^	BCODL-SDSC, FOLS, TSO, ARO, SRNN	Accuracy, Sensitivity, Specificity, F-Score, MCC	Benchmark Medical Image Dataset	The model attained secure data sharing and classification with a peak accuracy of 99.11%
Alamro et al.^14^	BHS-ALOHDL, ALO-FSS, CNN-LSTM, FPA	Accuracy, Precision, Recall, F-Score, AUC-Score	Two Benchmark Dataset	The method enhanced intrusion detection accuracy and processing speed in IoT healthcare systems
Mohananthini, Rajeshkumar, and Ananth^15^	BGJOA-DLSMTD, GJOA, Homomorphism Encryption, BC, BOA, DBN, CapsNet-based Feature Extraction	Accuracy, Sensitivity, Specificity, F-Score, MCC	ISIC Dataset	The method attained superior performance in medical image encryption, secure transmission, and disease diagnosis
Qu et al.^16^	Quantum BC and NN, Controlled Quantum Walk Hash Function, Quantum Authentication Protocol, HQCNN	TR/TS Accuracy, Detection Stability	Standard Dataset	HQCNN attains high accuracy and stability, outperforming classical CNN with greater robustness to quantum noise
Naveen, Dhivya, and Jenefa^17^	DenseNet Architecture, BC, TL, Bottleneck and Transition Layers	Accuracy, Precision, Recall, F1-Score	Dermoscopic Image Dataset	DenseNet and BC integration improves melanoma detection accuracy and efficiency
Kateb et al.^18^	ESHCS-DLJSO, Min–Max Normalization, BFOA, CNN-LSTM-Attention, JSO	Accuracy	IoT Healthcare Security Dataset	The model attains a superior accuracy of 99.43%, enhancing disease detection and IoT healthcare security
Rahal et al.^19^	DL, BC, Model Ensembling, Secure Model Sharing	Accuracy	Breast Cancer Dataset	The BC-based ensembling approach improves diagnostic accuracy and protects patient privacy
Hota et al.^20^	CNN	Accuracy, Precision, Recall, F1-Score	Standard Dataset	The system ensures privacy and prevents data breaches while attaining 92% accuracy in diagnosis
Rokade and Mishra^21^	BC, LeNet, TCIO, DeepJoint Segmentation with Kumar-Hassebrooks Distance	Accuracy, True Positive Rate, True Negative Rate, False Negative Rate, False Positive Rate	Benchmark Dataset	The model attained high accuracy and a robust detection rate for classifying skin diseases
Islam et al.^22^	ML, Predictive Analytics, AI, DL	Diagnostic Accuracy, Disease Prediction, Resource Optimization	Commonly Used Public Datasets	ML and AI improve disease detection and healthcare efficiency, but privacy, security, and bias issues remain
Shammi et al.^23^	ML, DL, BC	Disease Diagnosis, Data Security, Patient Privacy	Public Dataset	AI improves diagnosis and monitoring, while BC secures data and privacy
Matlo et al.^24^	PoWV-XAI, BC	Delay Control, Energy Consumption, Cost Efficiency, Security Validation	Various Dataset	PoWV-XAI enhances explainability and optimizes delay, energy, cost, and security
Khan and Jilani^25^	RSA, Blowfish Encryption, BC, ANN, CNN, RNN	Accuracy, Security	Heart Disease Data	The model ensures secure, accurate heart disease prediction (0.9941) while preserving privacy in cloud healthcare systems
Kulkarni et al.^26^	BC-based IoT, Privacy Matrix Generation, AHPO, Deep Maxout Network, SpinalNet	Accuracy, True Positive Rate, True Negative Rate	Healthcare Data	The model ensures private, accurate healthcare classification with high TPR and TNR
Gupta, Kumar, and Gupta^27^	FL, BC, DenseNet-201, FedAvg Aggregation, IPFS for Storage	Accuracy, Precision, Recall, F1-Score	Heterogeneous Datasets	The FL-BC framework detects lung disease with 90% accuracy while preserving data privacy
Ahmed et al.^28^	DL, PSO-BioBERT, Named Entity Recognition, Relation Extraction	NER and RE Accuracy, NER and RE F1-Score, Accuracy Gain over Bi-LSTM and BERT	Medical Dataset	The technique improves NER and RE performance, improving smart healthcare data analysis and decision-making
Dubey, Kapoor, and Saraswat^29^	Hybrid Learning Methods, Biological Feature Selection, BC-based Data Storage, Bidirectional LSTM, Time-series Data Categorization, Alzheimer’s Diagnosis	Multi-disease Prediction, Intrusion Detection, Alzheimer’s Diagnosis Accuracy, Image Segmentation Quality	EEG Data Dataset	The method improves early disease diagnosis and risk reduction using advanced algorithms and image analysis
Chougule et al.^30^	Entropy, Correlation Coefficient	Accuracy	Healthcare Dataset	The model enhances healthcare prediction accuracy and data security using advanced techniques
Al-Marridi, Mohamed, and Erbad^31^	BC, DRL, Multi-Agent Q-Learning, MDP, Computational resource optimization, Cooperative-competitive decision-making, QoS Optimization	Decision-making speed, Resource Utilization, Latency Minimization, Cost Minimization, Security Maximization, Qos Adherence	Simulated Healthcare Data	IP-HealthChain improves decision speed, mitigates latency, optimizes resources, enhances security, and cuts costs
Pradhan et al.^32^	ML, NN, HIoT, EHR, Security Enhancements, QoS	Security Improvement, QoS Enhancement, Accuracy of ML Models	Benchmark Dataset	ML applied to HIoT can improve security and QoS in EHR systems

The limitations of the existing approaches are data privacy, scalability, and the integration of multiple technologies such as BC and DL. Several models face issues with processing efficiency, particularly in large-scale healthcare systems and the management of computational resources, while BC ensures secure data storage and sharing. Furthermore, most DL models utilized in healthcare are highly dependent on large, high-quality datasets, which may not always be available due to privacy concerns and data fragmentation across various healthcare sectors. Additionally, a research gap exists in optimizing the collaboration between BC and DL for real-time healthcare applications, specifically in improving the scalability of BC-based data-sharing systems. The complexity of integrating BC with advanced DL techniques often results in performance bottlenecks and difficulties in maintaining real-time diagnosis. Another research gap is the limited exploration of hybrid models incorporating BC, DL, and other AI techniques, such as RL or FL, which could provide more efficient and secure solutions. Moreover, many studies have not adequately addressed interoperability issues between diverse healthcare systems, affecting seamless data exchange and collaboration. Lastly, there is also a requirement for better models to handle heterogeneous data sources in IoT-enabled healthcare systems while ensuring privacy and security.

## Materials and methods

**Fig. 1:**
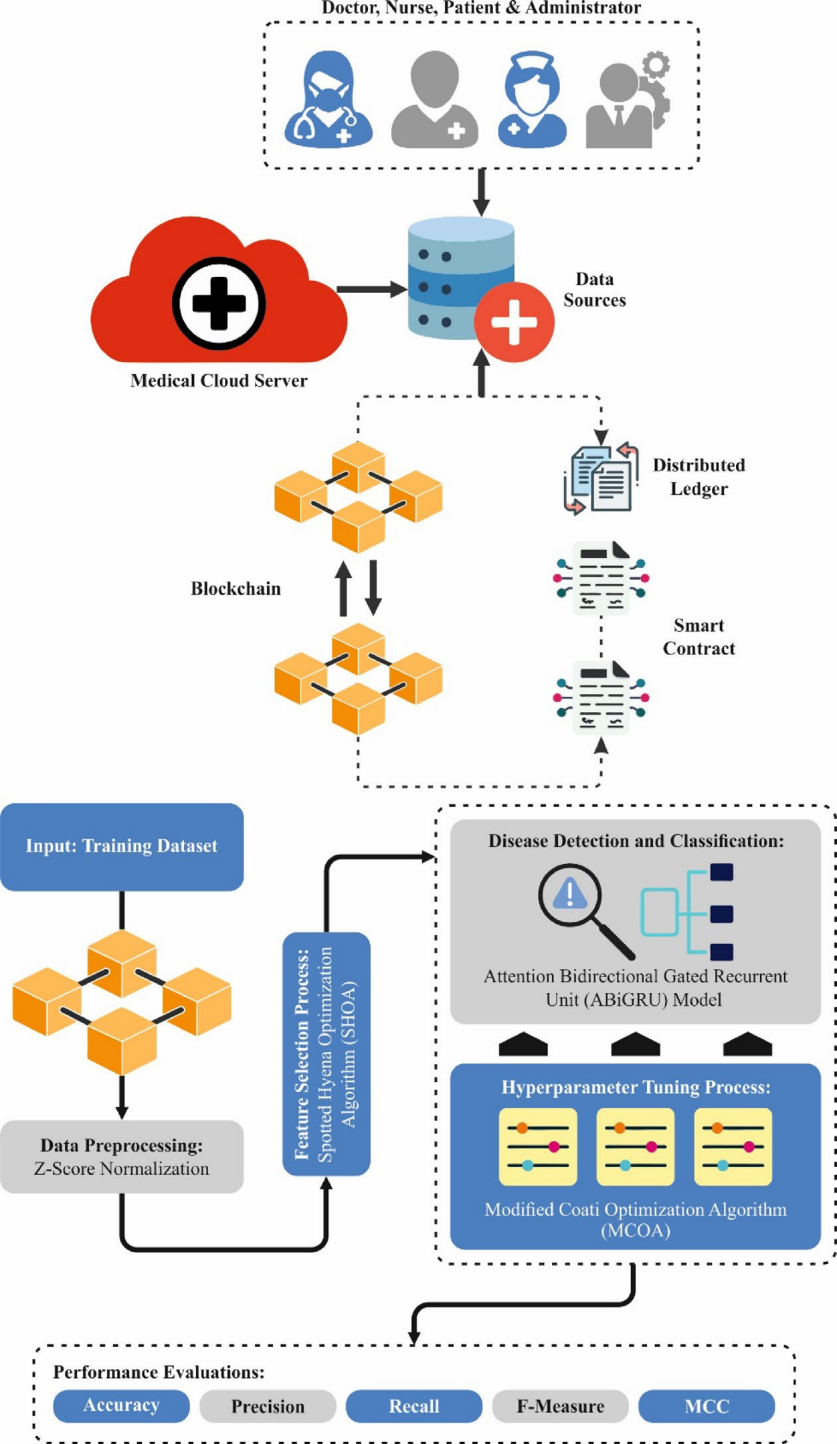
Workflow of MCODBC-HDDC method.

This manuscript proposes the MCODBC-HDDC method. The presented MCOBC-HDDC method provides an efficient and accurate diagnosis of disease utilizing a system based on DL techniques. To accomplish that, the MCODBC-HDDC model has different kinds of processes, as represented in Fig. 1.

### Blockchain (BC) in healthcare

BC provides very substantial use in healthcare information with the research of medical^33^. A healthcare organization can do this with the aid of BC, along with the clinical exploration and claim procedure. The chain that utilizes BC was employed to upsurge safety for the spread of patient data through numerous companies. Although BC benefits healthcare organizations, a few latent vulnerabilities contain scalability, privacy, and cost. BC tackles challenges like interoperability and safely allows information sharing among experts and patients. The BC intends to manage healthcare applications and must tackle security, data privacy, and scalability. BC utilizes cryptographic notations for its effectual functioning. The healthcare data gateway structure was intended to use BC, which aids patients in splitting their healthcare data without negotiating confidentiality and endorsing intelligence$.$ The BC was employed to hand over the health data safely through the distributed system. The architecture employs BC, where accord protocol was deployed to deliver the proof of authority. BC‐enabled intelligent IoT structure was proposed to utilize AI methods over the data firmly spread in the BC system. At the same time, there are manifold platforms and structures accessible for medical data organizations utilizing BC. However, a meta-heuristic technique is presented to learn from the obtainable data in the BC system. The idea behind the BC combination is to contain a proven learning resource. In a dynamic atmosphere, there is a greater chance of data management assaults, and the BC network might prohibit those.

### Data pre-processing

In the data preprocessing stage, the MCODBC-HDDC model employs Z-score normalization to standardize the data and improve performance^34^. This model is chosen for its efficiency in standardizing features by centring them around a mean of zero and a standard deviation of one. The model is appropriate when the dataset comprises features with diverse units or scales, ensuring that no single feature disproportionately affects the model. Unlike min–max scaling, which can be sensitive to outliers, Z-score normalization is more robust in handling extreme values. The model also improves convergence and stability, particularly for optimization-based DL techniques such as ABiGRU. Creating a uniform scale across features enhances the model’s learning efficiency and predictive performance. This makes it an appropriate and reliable choice over other normalization techniques in healthcare data applications.

${\boldsymbol{Z}}$‐score normalization is an essential method for feature scaling. Post-standardization, every feature part a standard scale, boasting mean $({\boldsymbol{\mu}})$ of 0 and SD $({\boldsymbol{\sigma}})$ of 1. This procedure significantly improves the precision of predictive methods.

1 $$x_{new} = \frac{x - \mu }{\sigma }$$

whereas, $x$ defines the new feature value, ${x}_{new}$ implies the standardized value, $\mu$ relates to the mean of a new feature, and $\sigma$ demonstrates the SD of the new feature.

### SHOA-based FS

**Fig. 2:**
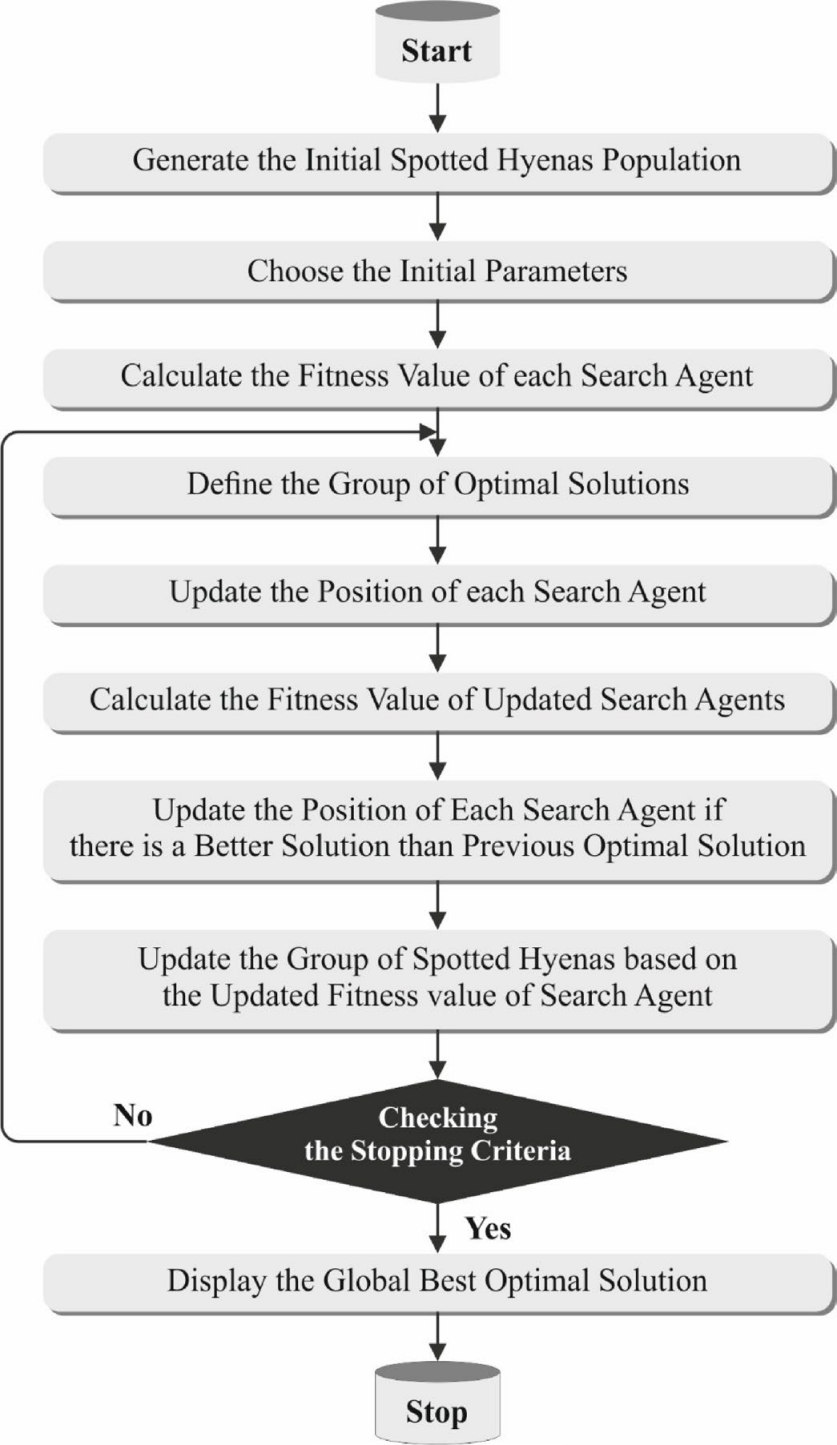
Workflow of SHOA technique.

For the optimal subset of features, the SHOA is utilized^35^. This model is chosen for its robustness in exploration and exploitation, which are crucial for detecting the most relevant features in high-dimensional healthcare data. The model is chosen for its intellectual hunting behaviour of hyenas. Conventional techniques such as genetic algorithms (GAs) or particle swarm optimization (PSO) exhibit limitations in navigating the search space, whereas this model shows excellency in navigation to avoid local optima. The model also dynamically balances global and local search strategies, resulting in more accurate and compact feature subsets. This mitigates computational load while maintaining or improving model accuracy. The adaptability and convergence speed of the model makes it appropriate for complex, nonlinear healthcare datasets where irrelevant or redundant features can degrade performance. Figure 2 illustrates the flow of the SHOA technique.

The SHOA aids as an ensuring optimizer device for tackling RDS optimizer tasks, delivering scalability, comfort of parameter tuning, and strong convergence while efficiently handling convergence problems and computation complexity. An adaptive stop condition is presented to stop iterations when no major development beyond a pre-defined threshold is kept in the last *K*th iteration, certifying that the technique ends correctly upon convergence or evades premature uncertainty when the function of the objective endures to progress. The SHOA is a nature-oriented optimizer method that appeals to stimuli from the features and behaviour of spotted hyenas. They are famous for their flexibility, endurance, and supportive searching behaviour, which are employed to resolve optimizer issues. The technique utilizes global and local search tactics and adjustable operators and parameters for increasing performance. The flexible and effective behaviour of hyenas stimulates its new method. The SHOA is an optimization technique that uses formulations to repeatedly hunt for the nearest global optimal solution to an assumed issue. The key steps of SHOA are mentioned below:

*Encircling prey*: The technique involves deliberating numerous hunt aspects and constantly upgrading the near global optimal location concerning the target. The mathematical method employed to signify this action is stated over an exact formulation, which can vary depending on the optimizer issue.

2 $$v_{h} \left( {j + 1} \right) = v_{p} \left( t \right) - Z*R_{hp}$$

$R_{hp}$ signifies the distance between a spotted hyena and its victim; $v_{p}$ denotes the location vector of prey, $v_{h}$ denotes the location vector of the spotted hyena. $j$ signifies the present iteration, $Y,$ and $Z$ represents the coefficient factor vectors $.$

3 $$Y = 2v_{r1}$$

4 $$Z = 2l*v_{r2} - l$$

5 $$l = 5 - \left( {Iter*\left( {\frac{5}{{Max_{iter} }}} \right)} \right)$$

Here, $$Iter=\mathrm{0,1},2,\dots ,{Max}_{Iter}.$$

In the given context, ${v}_{r1}$ and ${v}_{r2}$ are randomly produced vectors in the interval of $(\mathrm{0,1})$. Besides, the 1 value is linearly decreased from 5 to $0$ in an assumed range.

*Hunting*: The hunting tactic is portrayed as below:

6 $$R_{hp} = \left| {Y*v_{p,best} \left( j \right) - v_{h,best} \left( j \right)} \right|$$

7 $$R_{hp} = \left| {Y*v_{p} \left( j \right) - v_{h} \left( j \right)} \right|$$

8 $$v_{h,best} = v_{p,besi} - Z*R_{hp}$$

9 $$Op_{h} = v_{h,best} + v_{h,best + 1} + \cdots v_{{h,best + N_{h} }}$$

whereas, ${v}_{p,besi}$ represents the most favourable posture of spotted hyenas relative to the prey. ${v}_{h,best}$ signifies another position for the spotted hyena. The below-mentioned equation is employed to calculate the complete number of spotted hyenas, denoted as ${N}_{h}.$

10 $$N_{h} = C_{n} \left( {v_{p,best} ,v_{p,best + 2} , \ldots v_{p,best + G} } \right)$$

While, the randomly produced vector $G$ lies in the range of (0.5, 1). $n$ signifies the total amount of responses. $C_{n}$ indicates a range of $N_{h}$ best responses collected jointly.

*Attacking prey (exploitation)*: The procedure of prey attack is mentioned below in the mathematical formulation:

11 $$v_{h} \left( {x + 1} \right) = \frac{{C_{n} }}{{N_{h} }}$$

Here, ${v}_{h}(x+1)$ is in the control of protecting the finest response and adapting the positions of other origins depending upon the finest search element location.

*Search for prey (exploration)*: To discover the correct resolution, the $Z$ value should be higher or lesser than one. $Y$ is one more SHOA module, which aids with exploration. The $Y$ contains of produced value at random, which allocates random weight to the victim. In the vector of *Y*, the elements that are larger than 1 are assumed larger priority than those lesser than 1.

*Adaptive stopping criterion*: This step is combined into the major circle of SHOA, naturally behind the development of iteration is observed and upgraded. This check is executed at the finale of every iteration to define whether to stop additional iteration. By integrating this criterion, we can enhance its efficacy and stop excessive calculation in the objective function.

If $\left|f\left({v}_{h,best}\left(j\right)\right)-f\left({v}_{h,best}\left(j-1\right)\right)\right|<e$ for $k$ consecutive iteration, then the algorithm ends and yields a response. Here $\epsilon$ denotes a smaller value of the positive threshold, $f\left({v}_{h,best}\left(j\right)\right)$ represents the value of the objective function at $jth$ iteration, $k$ signifies the amount of consecutive iterations. The condition verifies if the development drops under a threshold $\epsilon$ over a specified amount of consecutive iterations, representing latent convergence or absence of major development. By overwhelming down the steps drawn in the algorithm, it provides clarity and enables the ability of the developed technique. This method creates the execution of the SHOA handier, certifying that every step is well-defined. During the SHOA, the purposes can be combined as a single objective that an existing weight recognizes all the objective positions. In this case, it is implemented a fitness function (FF) that integrates both ideas of FS as depicted in Eq. (12).

12 $$Fitness\left( X \right) = \alpha \cdot E\left( X \right) + \beta *\left( {1 - \frac{\left| R \right|}{{\left| N \right|}}} \right)$$

Here, $Fitness(X)$ defines the fitness value of subset $X,$ $E(X)$ demonstrates the classifier value of errors by employing the preferred features from the *X* subset, $|R|$ and $|N|$ signifies the preferred feature counts and the number of new features from the database, $\alpha$ and $\beta$ implies the weighted of classifier error and the ratio of reduction.

### Disease detection using ABiGRU classifier

**Fig. 3:**
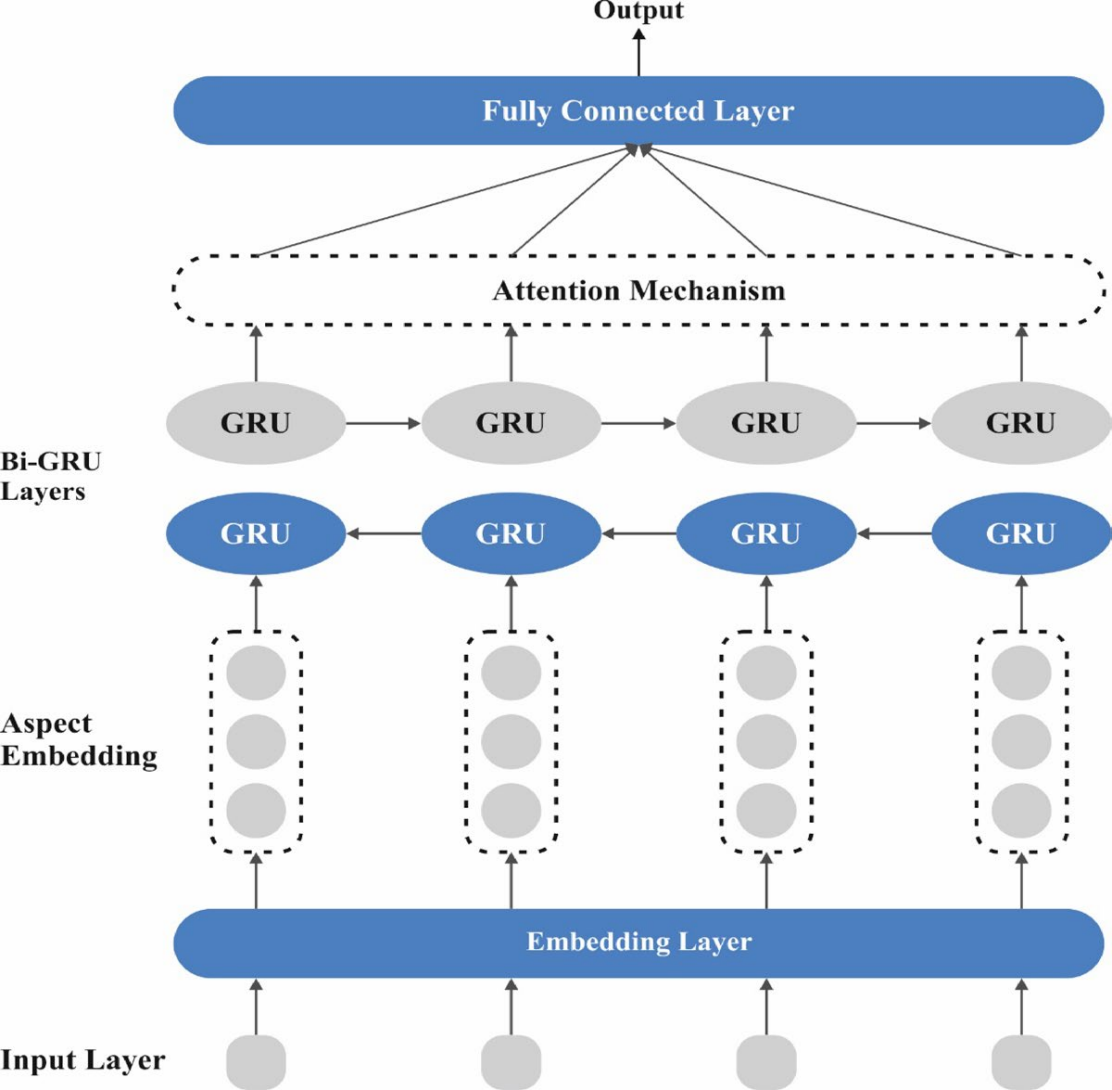
Architecture of ABiGRU model.

Besides, the ABiGRU method is employed for disease recognition and classification^36^. This method is chosen due to its ability to effectually capture temporal dependencies and contextual data in sequential healthcare data. Unlike standard RNNs or unidirectional GRUs, the bidirectional structure processes information in both forward and backward directions, improving the comprehension of complex patterns. The integrated attention mechanism enhances performance by allowing the model to focus on the most relevant features in the input sequence. Compared to CNNs or conventional ML classifiers, ABiGRU performs better on time series or structured health records. Its efficiency, lower computational cost than LSTMs, and enhanced interpretability make it ideal for accurate and explainable disease classification. Figure 3 depicts the infrastructure of ABiGRU.

As an alternative to GRU, RNNs and LSTM represent a gate mechanism to avoid the gradient vanishing and explosion difficulties in traditional RNNs. Based on LSTM, GRU improves the gate structure and facilitates input and output, and forget gates are converted into update and reset gates that upsurge computational efficacy and reduce the number of parameters.

13 $$r_{t} = \sigma \left( {W_{r} \cdot \left| {h_{t - 1} , x_{t} } \right|} \right)$$

14 $$z_{t} = \sigma \left( {W_{z} \cdot \left| {h_{t - 1} , x_{t} } \right|} \right)$$

15 $$\tilde{h}_{t} = \tanh \left( {W \cdot \left| {r_{t}^{*} h_{t - 1} , x_{t} } \right|} \right)$$

16 $$h_{t} = (1 - z_{t} )^{*} h_{t - 1} + z_{t}^{*} \tilde{h}_{t}$$

The reset gate ${r}_{t}$ describes how much past data is to be disregarded. The update gate ${Z}_{t}$ creates the number of preceding data, which will be distributed on later. The sigmoid function can be expressed by $\sigma ;{x}_{t}$ denotes the input at $t$ time the weighted matrices of the hidden layer (HL), update gate, and reset gate are $W,{W}_{z}$, and ${W}_{r}$ respectively; ${h}_{t-1}$ signifies the state of HL at $t-1$ time; ${\widetilde{h}}_{t}$ mentions to the sum of the past and current state of HL at time $t$; and ${h}_{t}$ represents the HL output. The unidirectional GRU is frequently outputted from front to back and can’t take the involvement of succeeding information to the semantics. Bi-GRU attains text data from both directions and utilizes it as the final output.

The current input ${x}_{t}$ states the current Bi-GRU state of HL; the reverse and the forward state of HL outputs at $t-1$ time are $\overleftarrow{{h}_{t-1}}$ and $\overrightarrow{{h}_{t-1}}$. The Bi‐GRU can be separated into dual one-way GRUs, and the Bi‐GRU state of HL at $t$ time is obtained by the weighting sum of ${\overleftarrow{h}}_{t-1}$ and ${\overrightarrow{h}}_{t-1}$:

17 $$h_{t} = w_{t} \cdot GRU\left( {x_{t} ,\vec{h}_{t - 1} } \right) + v_{t} \cdot GRU\left( {x_{t} ,\mathop{h}\limits^{\leftarrow} _{t - 1} } \right) + b_{t}$$

Here, the GRU() function signifies the input word vector non‐linear conversion and encodes them into the corresponding state of HL. $w_{t}$ and $v_{t}$ are the weights compared to the $\vec{h}_{t}$ and $\mathop{h}\limits^{\leftarrow} _{t}$ (HLs), respectively, and $b_{t}$ means the bias to the state of HL at $t$ time.

While the word vectors $x_{1} ,x_{t - 1} ,x_{t}$, and $x_{n}$ output the feature vectors of HL $h_{1} ,h_{t - 1} ,h_{t}$, and $h_{n}$ by the Bi‐GRU and multiply it with the $a_{1} ,a_{t - 1} ,a_{t}$, and $a_{n}$ weighted coefficients and gather them as an output $V$ of the attention layer and it is computed as.

18 $$e_{\iota } = v_{\iota } \tanh \left( {w_{\iota } h_{\iota } + b_{\iota } } \right)$$

19 $$a_{i} = \frac{{ \exp \left( {e_{i} } \right)}}{{\mathop \sum \nolimits_{j = 1}^{n} 1\left( {e_{j} } \right)}}$$

20 $$V = \mathop \sum \limits_{i = 1}^{t} a_{i} h_{i}$$

where the state vector of HL can be denoted as ${e}_{\iota }$ is. ${w}_{\iota }$ and ${v}_{\iota }$ specify the weight coefficient matrices. ${b}_{\iota }$ states to the bias. The attention score ${a}_{\iota }$ defines the word significance to the sentence. $V$ symbolizes the output vector of the weighted amount of all the words.

### MCOA-based parameter tuning

**Fig. 4:**
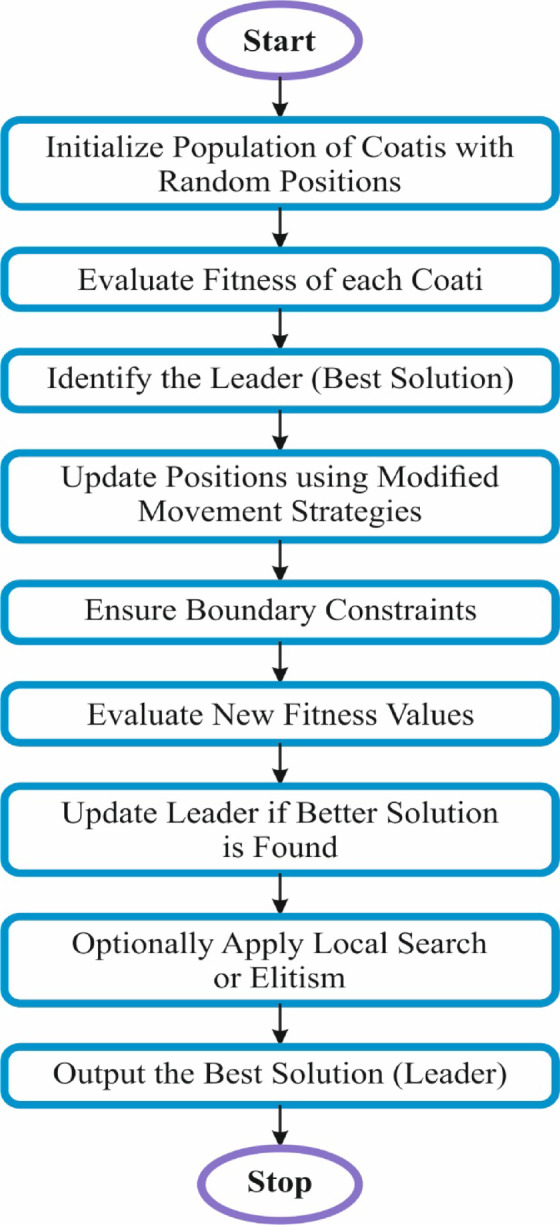
Flow of the MCOA technique.

Eventually, the hyperparameter selection of the ABiGRU model is performed by implementing the MCOA technique^37^. This model is chosen due to its improved capability to balance exploration and exploitation during the search process. Inspired by the natural behaviour of coatis, MCOA introduces modifications that improve convergence speed and solution accuracy compared to standard optimization methods like grid search, random search, or even conventional metaheuristics such as GA and PSO. It efficiently navigates complex hyperparameter spaces, mitigating the risk of getting trapped in local optima. The model’s adaptability and dynamic search capability make it specifically effectual for DL models, where optimal parameter selection is significant for optimizing performance. Its usage results in enhanced model generalization, reduced overfitting, and improved prediction accuracy in disease detection tasks. Figure 4 demonstrates the working flow of the MCOA method.

The COA is a new optimization technique stimulated by the coatis’ evasion and predatory behaviours. The method is separated into dual stages. For example, the first stage pretends the coatis’ catching iguanas, which signifies the exploration behaviour; the second stage pretends the coatis’ behaviour of evading predators, which represents the exploitation behaviour.

#### Hunting and attacking strategy

When coatis search iguanas, they use to perform in clusters. This hunting tactic splits the population of coati into dual parts, each one hunting for prey. In COA, it is highly expected that 50 percent of coatis climb trees to catch iguanas, while the remaining stay on the ground to catch the falling iguanas. The mathematical formulation is mentioned below:

21 $$X_{i}^{P1} :x_{i,j}^{P1} = x_{i,j} + r \cdot \left( {Xbest_{j} - I \cdot x_{i,j} } \right)$$

where ${x}_{i,j}^{P1}$ signifies the $j$th dimension of the $i$th coati in the first phase; $Xbes{t}_{j}$ signifies the $j$th dimension of the finest solution; $I$ denotes a randomly produced values {1, 2}. When the iguanas fall to the ground, the coatis endure to search the iguanas.

22 $$X^{G} :x_{j}^{G} = lb_{j} + r \cdot \left( {ub_{j} - lb_{j} } \right)$$

where ${X}^{G}$ signifies the produced location at random of the iguana; $l{b}_{j}$ and $u{b}_{j}$ signifies the lower and upper limit of the $jth$ dimension, respectively; $r$ means the randomly generated value in $[\mathrm{0,1}].$

23 $$X_{i}^{P1} :x_{i,j}^{P1} = \left\{ {\begin{array}{*{20}l} {x_{{i_{^{\prime}} j}} + r \cdot \left( {X_{j}^{G} - I.x_{i,j} } \right),\quad F_{XG} < F_{i} } \hfill \\ {xi,j + r \cdot \left( {x_{i,j} - X_{j}^{G} } \right), \quad else} \hfill \\ \end{array} } \right\}$$

Here $I$ denotes a randomly produced value of {1, 2}; $r$ means the generated value at random in $\left[\mathrm{0,1}\right];$ ${F}_{{X}^{G}}$ and ${F}_{i}$ signifies the value of the cost function of the recently produced iguana place and original population, respectively; $r$ means a randomly generated value in the interval of $\left(\mathrm{0,1}\right);$ and ${x}_{i,j}$ signifies the $jth$ dimension of $ith$ individual data. The novel location will be upgraded in the population as presented in Eq. (24).

24 $$X_{i} = \left\{ {\begin{array}{*{20}l} {X_{i}^{P1} ,} \hfill & {F_{i}^{P1} < F_{j} } \hfill \\ {X_{i} ,} \hfill & {else.} \hfill \\ \end{array} } \right.$$

where ${X}_{i}$ signifies the location of $ith$ coatis; ${X}_{i}^{P1}$ epitomizes the location that the coatis noticed in stage 1; $F$ means the cost value consistent with the location of the coatis.

#### The procedure of escaping from predators

When coatis are followed by hunters, they arbitrarily jump to nearby locations to avoid them. This procedure is pretend by the below-mentioned formulation:

25 $$lb_{j}^{local} = \frac{{lb_{j} }}{t}, ub_{j}^{local} = \frac{{ub_{j} }}{t}$$

26 $$X_{i}^{P2} : x_{i,j}^{P2} = x_{i,j} + \left( {1 - 2r} \right) \cdot \left( {lb_{j}^{local} + r \cdot \left( {ub_{j}^{local} - lb_{j}^{local} } \right)} \right)$$

where $l{b}_{j}^{local}$ and $u{b}_{j}^{local}$ represents the escape range and ${X}_{i}^{P2}$ signifies the location after the coatis were harassed.

27 $$X_{i} = \left\{ {\begin{array}{*{20}l} {X_{i}^{P2} ,} \hfill & {F_{i}^{P2} < F_{i} } \hfill \\ {X_{i} ,} \hfill & {else.} \hfill \\ \end{array} } \right.$$

Here, ${X}_{i}$ means the location of the $ith$ coatis; ${X}_{i}^{P2}$ signifies the location that the coatis noticed in stage 2; and $F$ represents the cost value.

In the predation stage, the COA evades dividing the population into dual portions: the first part upgrades individuals directed by the finest value, and the other part upgrades the technique by utilizing randomly generated values. The outcomes perceived by these dual parts are dissimilar, but the technique does not completely use this dissimilar information. The COA’s model of examining the finest solution is quite easy, which might not be adequate to get higher‐quality routes. Dynamic opposite learning (DOL) is an enhanced approach that depends upon opposition‐based learning (OBL). The mathematical formulation is mentioned below:

28 $$x^{dol} = x + r^{1} \left( {r^{2} x^{o} - x} \right)$$

29 $$x^{o} = a + b - x$$

whereas ${x}^{o}$ denotes an opposite operator, $a$ represents the lower limit, $b$ signifies the upper limit. After the population finishes its respective predation, the remaining population does differential DOL, and its upgrade formulation is mentioned below:

30 $$X_{i}^{new} = X_{i} + r_{c} \left( {r_{c} \left( {lowerbound + upperbound - X_{i}^{op} } \right) - X_{i}^{op} } \right)$$

Here, ${X}_{i}^{1}$ corresponds to the $ith$ individual in the pre half population. When upgrading the $ith$ individual in ${X}_{i}^{2}$, the equivalent ${X}_{i}^{op}$ denotes the $ith$ individual in the later half population. When upgrading the $ith$ individual in ${X}^{2}$, the equivalent ${X}_{i}^{op}$ represents the $ith$ individual in the pre half population. ${r}_{c}$ denotes a randomly generated value. The fitness choice is a great influence the effectiveness of MCOA. The parameter tuning contains the encoded method for evaluating the effectiveness of candidate outcomes. In this case, the MCOA assumes that accuracy as a primary condition to plan FF that is expressed as:

31 $$Fitness = \max \left( P \right)$$

32 $$P = \frac{TP}{{TP + FP}}$$

where FP and TP define the false and true positive values.

#### Experimental result and analysis

This section examines the performance validation of the MCODBC-HDDC model under the HD dataset^38^. The technique is simulated using Python 3.6.5 on a PC with an i5-8600k, 250GB SSD, GeForce 1050Ti 4GB, 16GB RAM, and 1TB HDD. Parameters include a learning rate of 0.01, ReLU activation, 50 epochs, 0.5 dropouts, and a batch size of 5. The dataset comprises 303 samples under two classes, as shown in Table 2. Also, the dataset contains 14 features, including age, sex, cp, trestbps, chol, fbs, restecg, thalach, exang, oldpeak, slope, ca, thal, and num. From these, nine features were selected: age, sex, cp, trestbps, fbs, exang, oldpeak, slope, and num.

**Table 2:** Details of the dataset.

Class	No. of instances
Presence	165
Absence	138
Total instances	303

**Fig. 5:**
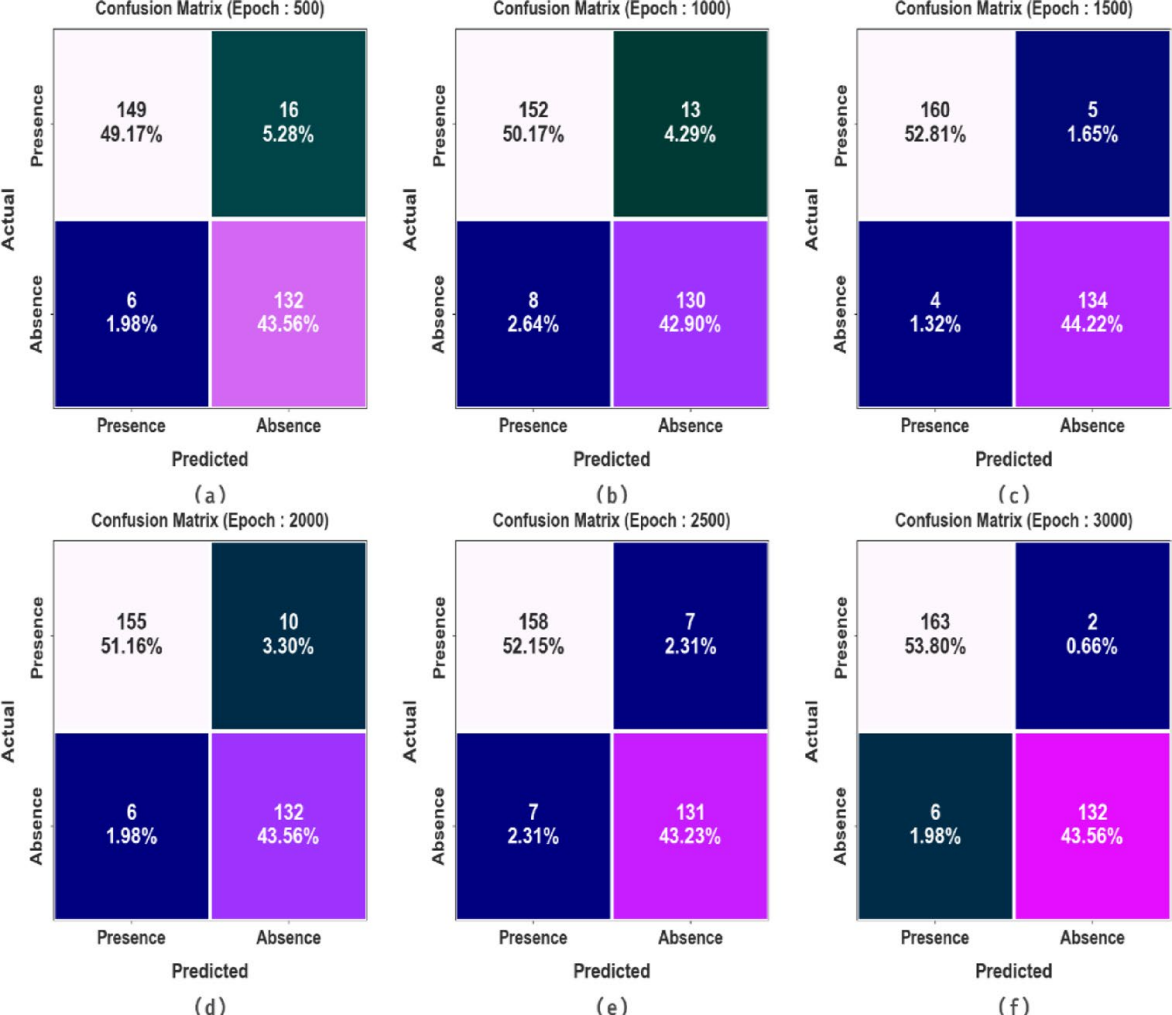
Confusion matrices of MCODBC-HDDC model (**a**–**f**) Epochs 500–3000.

Figure 5 illustrates the confusion matrices achieved by the MCODBC-HDDC methodology under 500–3000 epochs. The outcomes implied that the MCODBC-HDDC model effectively recognizes the presence and absence of instances under all classes.

**Fig. 6:**
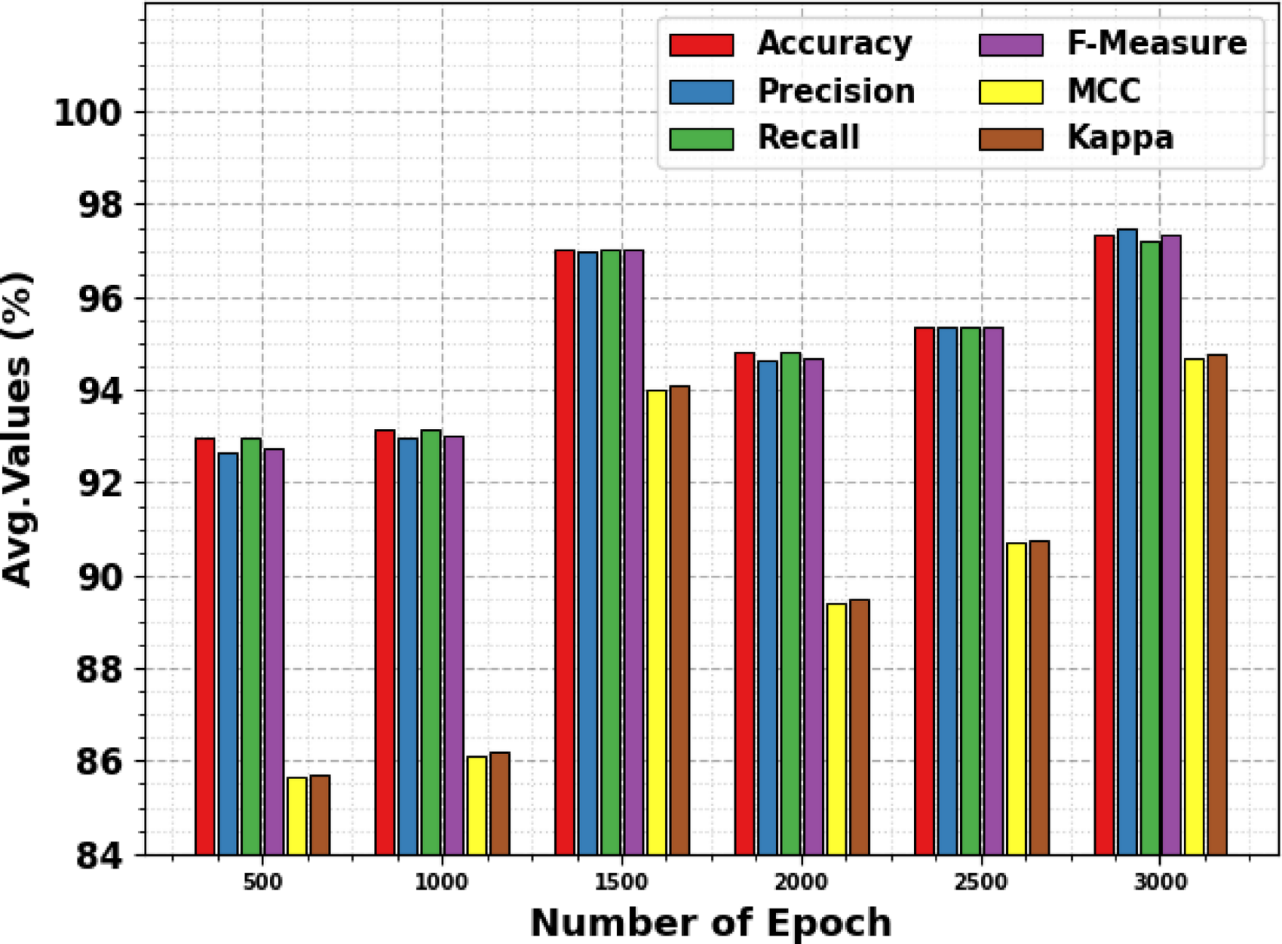
Overall classification outcome of MCODBC-HDDC technique under 500–3000 epochs.

Table 3 and Fig. 6 indicate the overall classification outcomes of the MCODBC-HDDC technique. The results stated that the MCODBC-HDDC technique properly recognized varied classes. With 500 epochs, the MCODBC-HDDC technique presents an average accu_y, prec_n, reca_l, F_measure, MCC, and Kappa of 92.98%, 92.66%, 92.98%, 92.72%, 85.64%, and 85.70%, correspondingly. Also, with 2000 epochs, the MCODBC-HDDC technique attains an average accu_y, prec_n, reca_l, F_measure, MCC, and Kappa of 94.80%, 94.62%, 94.80%, 94.69%, 89.41%, and 89.49%, correspondingly. Besides, with 3000 epochs, the MCODBC-HDDC approach attains an average accu_y, prec_n, reca_l, F_measure, and MCC of 97.36%, 97.48%, 97.22%, 97.33%, 94.70%, and 94.77%, subsequently.

**Table 3:** Overall classification outcome of MCODBC-HDDC technique under 500–3000 epochs.

Classes	${\mathrm{Accu}}_{\mathrm{y}}$	${\mathrm{Prec}}_{\mathrm{n}}$	${\mathrm{Reca}}_{\mathrm{l}}$	${\mathrm{F}}_{\mathrm{Measure}}$	MCC	Kappa
Epoch-500						
Presence	90.30	96.13	90.30	93.13	85.64	85.70
Absence	95.65	89.19	95.65	92.31	85.64	85.70
Average	92.98	92.66	92.98	92.72	85.64	85.70
Epoch-1000						
Presence	92.12	95.00	92.12	93.54	86.12	86.19
Absence	94.20	90.91	94.20	92.53	86.12	86.17
Average	93.16	92.95	93.16	93.03	86.12	86.18
Epoch-1500						
Presence	96.97	97.56	96.97	97.26	94.02	94.10
Absence	97.10	96.40	97.10	96.75	94.02	94.08
Average	97.04	96.98	97.04	97.01	94.02	94.09
Epoch-2000						
Presence	93.94	96.27	93.94	95.09	89.41	89.49
Absence	95.65	92.96	95.65	94.29	89.41	89.49
Average	94.80	94.62	94.80	94.69	89.41	89.49
Epoch-2500						
Presence	95.76	95.76	95.76	95.76	90.69	90.75
Absence	94.93	94.93	94.93	94.93	90.69	90.75
Average	95.34	95.34	95.34	95.34	90.69	90.75
Epoch-3000						
Presence	97.36	96.45	98.79	97.60	94.70	94.77
Absence	97.36	98.51	95.65	97.06	94.70	94.78
Average	97.36	97.48	97.22	97.33	94.70	94.77

**Fig. 7:**
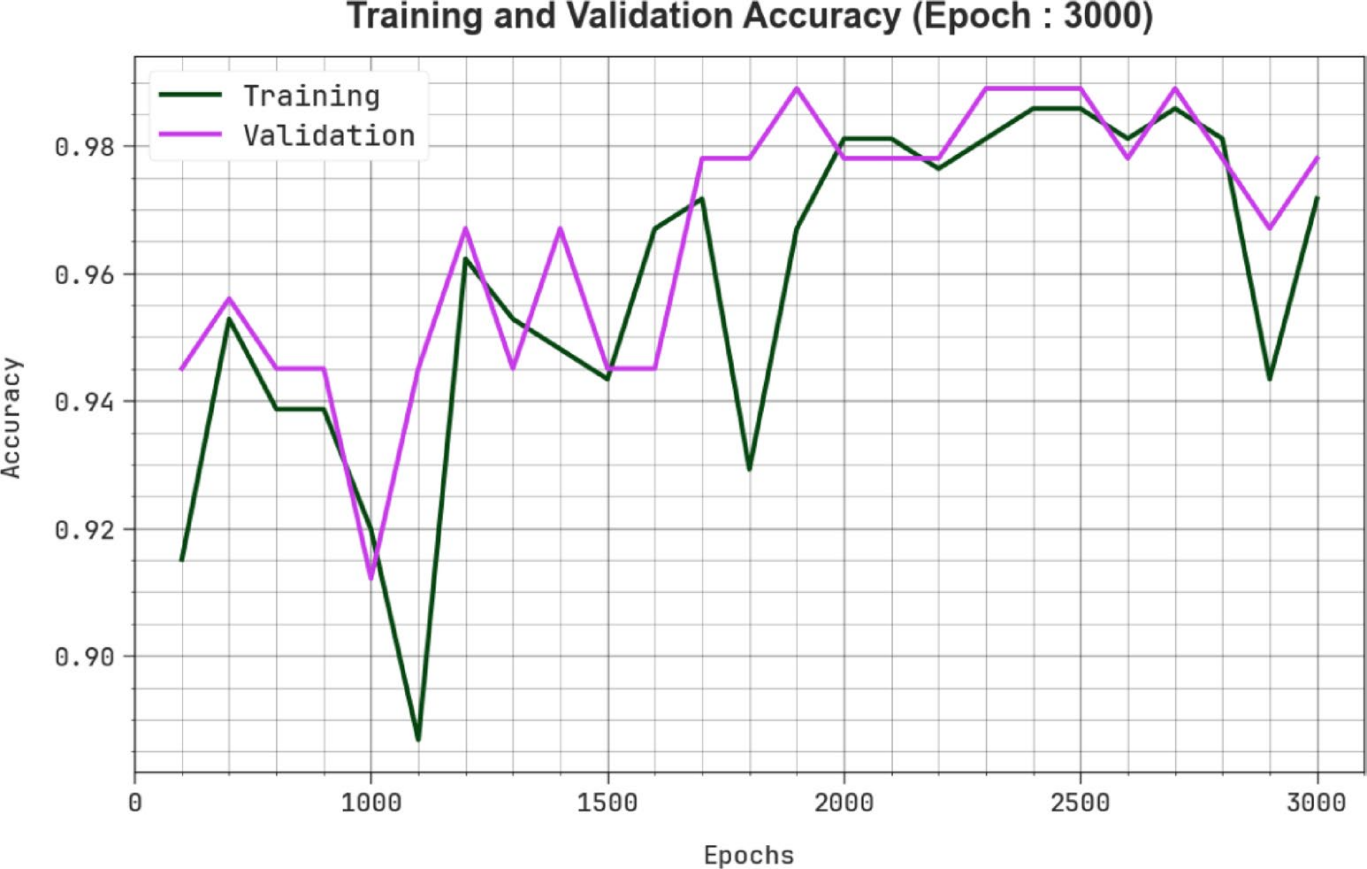
$Acc{u}_{y}$ curve of MCODBC-HDDC model under 3000 epochs.

In Fig. 7, the TRA $acc{u}_{y}$ (TRAAC) and validation $acc{u}_{y}$ (VLAAC) outcomes of the MCODBC-HDDC approach are established. The $acc{u}_{y}$ values are computed under intervals of 0–3000 epochs. The result showed that the TRAAC and VLAAC values exhibit an increasing tendency, which informed the proficiency of the MCODBC-HDDC technique, with improved outcomes at various iterations.

**Fig. 8:**
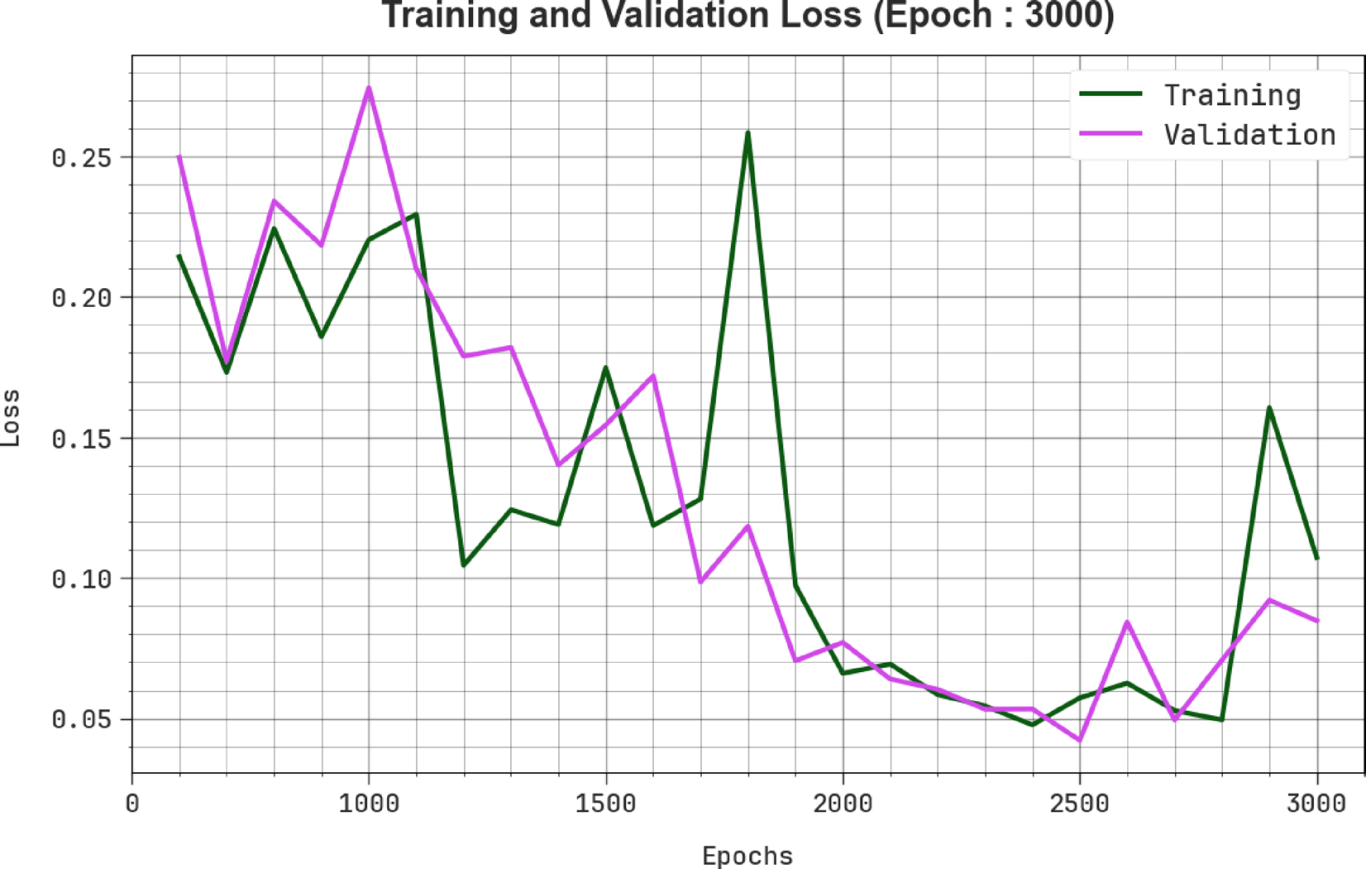
Loss curve of MCODBC-HDDC model under 3000 epochs.

Figure 8 illustrates the MCODBC-HDDC technique’s TRA loss (TRALS) and VLA loss (VLALS) graph. The loss values are designed under intervals of 0–3000 epochs. The TRALS and VLALS outcomes exemplify a reducing tendency, which indicates the approach’s ability to balance a trade-off between generalized and data fitting.

**Fig. 9:**
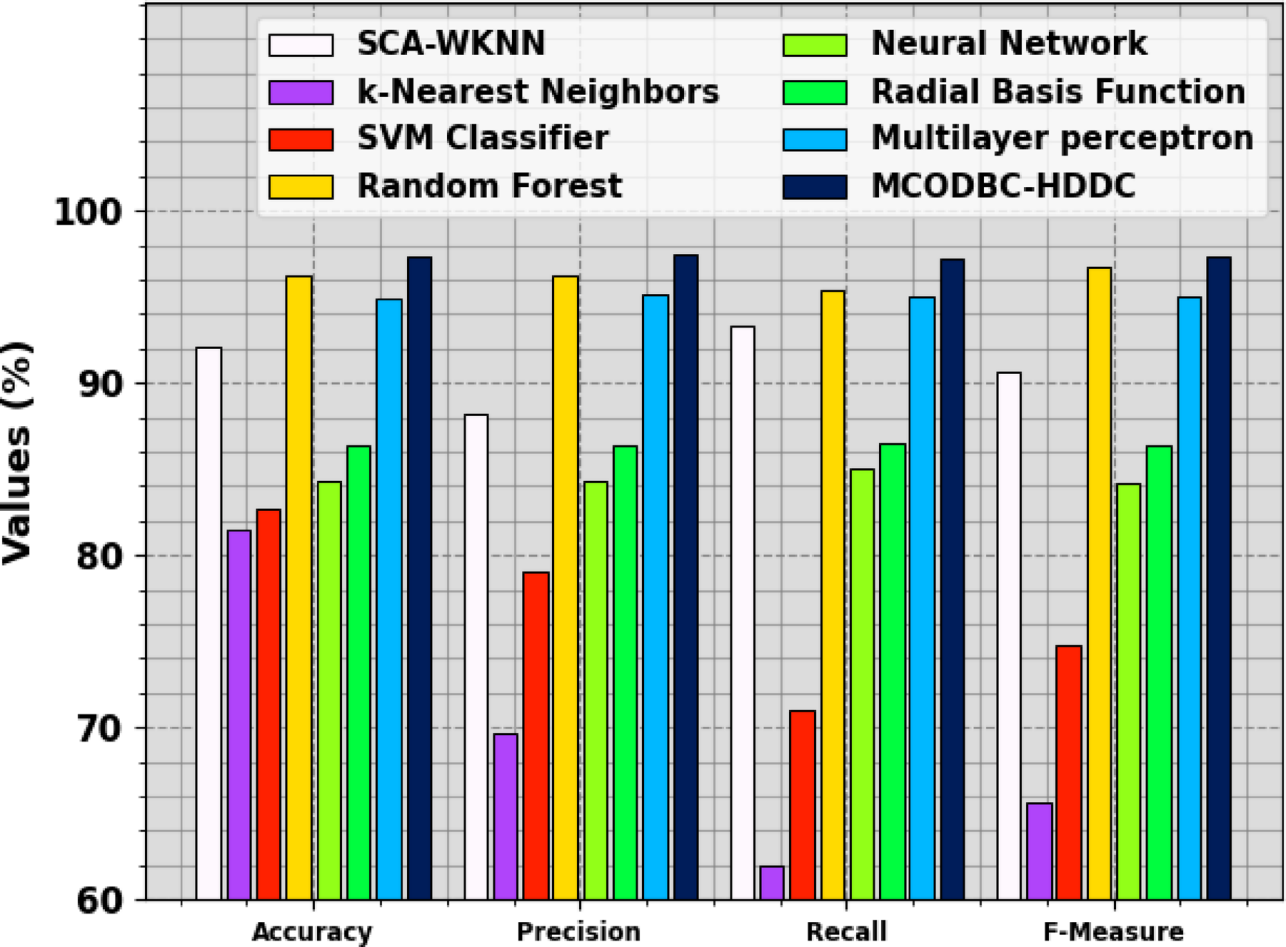
Comparison analysis of MCODBC-HDDC approach with existing methods.

To exemplify the better performance of the MCODBC-HDDC approach, a brief comparison study is made in Table 4 and Fig. 9^18,39^. The table values represented that the k-nearest neighbours (kNN), SVM, and NN approaches illustrated lesser classification performances. Likewise, the radial basis function (RBF), SCA-WKNN, and multilayer perceptron (MLP) approaches have tried to realize somewhat closer classification outcomes. Additionally, the random forest (RF) method has exhibited reasonable outcomes with $acc{u}_{y}$ of 96.28%, $pre{c}_{n}$ of 96.28%, $rec{a}_{l}$ of 95.37%, and ${F}_{measure}$ of 96.68%. However, the MCODBC-HDDC methodology determines promising performance with $acc{u}_{y}$ of 97.36%, $pre{c}_{n}$ of 97.48%, $rec{a}_{l}$ of 97.22%, and ${F}_{measure}$ of 97.33%.

**Table 4:** Comparative outcome of MCODBC-HDDC approach with existing methods.

Methodology	${\mathrm{Accu}}_{\mathrm{y}}$	${\mathrm{Prec}}_{\mathrm{n}}$	${\mathrm{Reca}}_{\mathrm{l}}$	${\mathrm{F}}_{\mathrm{Measure}}$
SCA-WKNN	92.13	88.21	93.27	90.66
kNN	81.49	69.70	62.00	65.62
SVM Classifier	82.70	79.00	71.03	74.80
RF	96.28	96.28	95.37	96.68
NN	84.33	84.33	85.01	84.13
RBF	86.35	86.35	86.44	86.35
MLP	94.96	95.16	95.06	95.06
MCODBC-HDDC	97.36	97.48	97.22	97.33

**Fig. 10:**
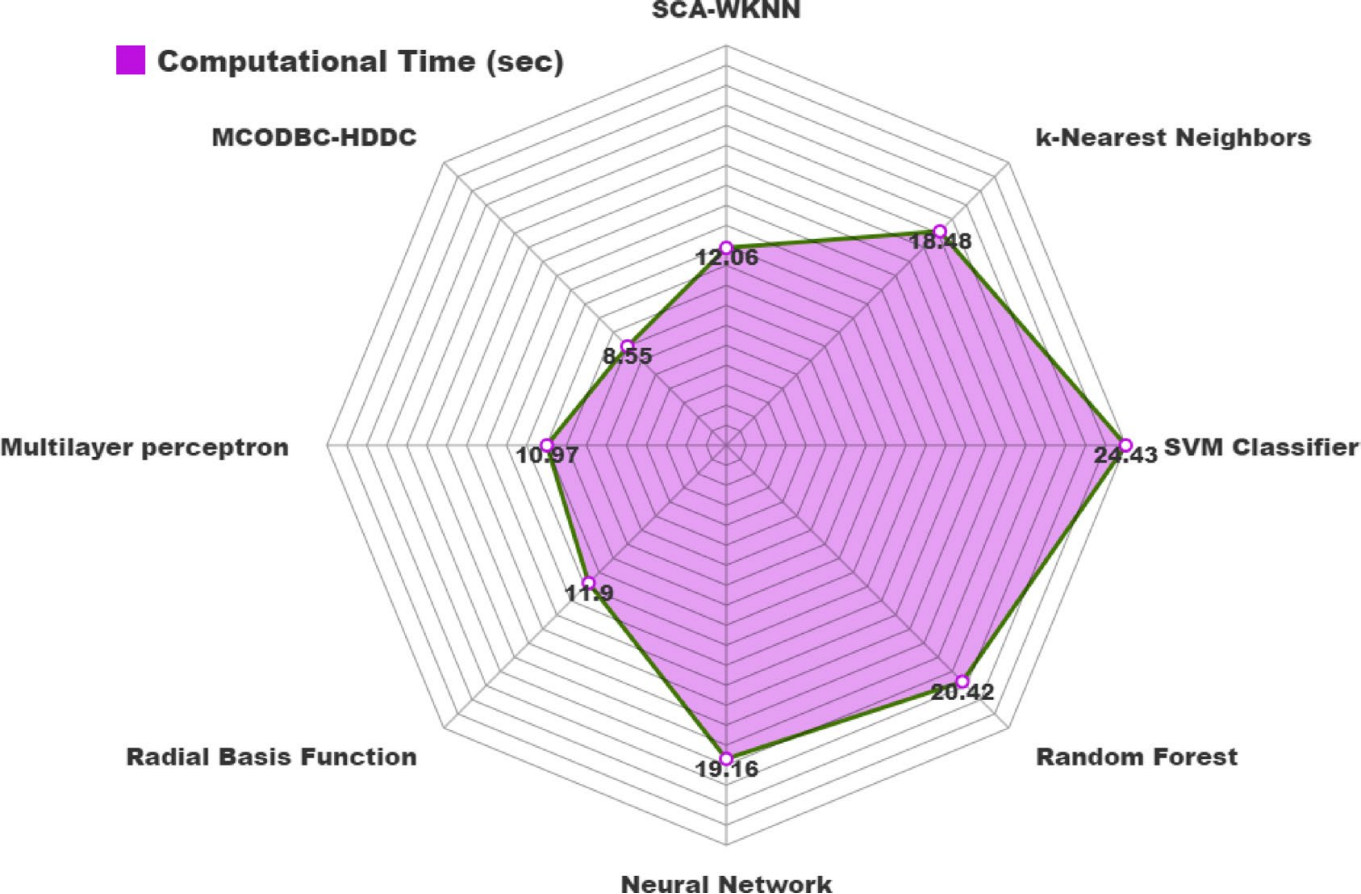
CT outcome of MCODBC-HDDC technique with other algorithms.

The computational time (CT) of the MCODBC-HDDC approach can be compared with existing models in Table 5 and Fig. 10. The outcomes highlighted that the SVM, RF, and NN methods have obtained worse performance with higher CT of 24.43s, 20.42s, and 19.16s, correspondingly. Followed by, the KNN, SCA-WKNN, and RBF systems have reported closer CT values of 18.48s, 12.06s, and 11.90s, correspondingly. In the meantime, the MLP model has managed to report a considerable CT of 10.97s. Nevertheless, the MCODBC-HDDC technique demonstrated superior performance with a lower CT of 8.55s.

**Table 5:** CT outcome of MCODBC-HDDC technique with existing models.

Methodology	CT (sec)
SCA-WKNN	12.06
kNN	18.48
SVM Classifier	24.43
RF	20.42
NN	19.16
RBF	11.90
MLP	10.97
MCODBC-HDDC	8.55

**Fig. 11:**
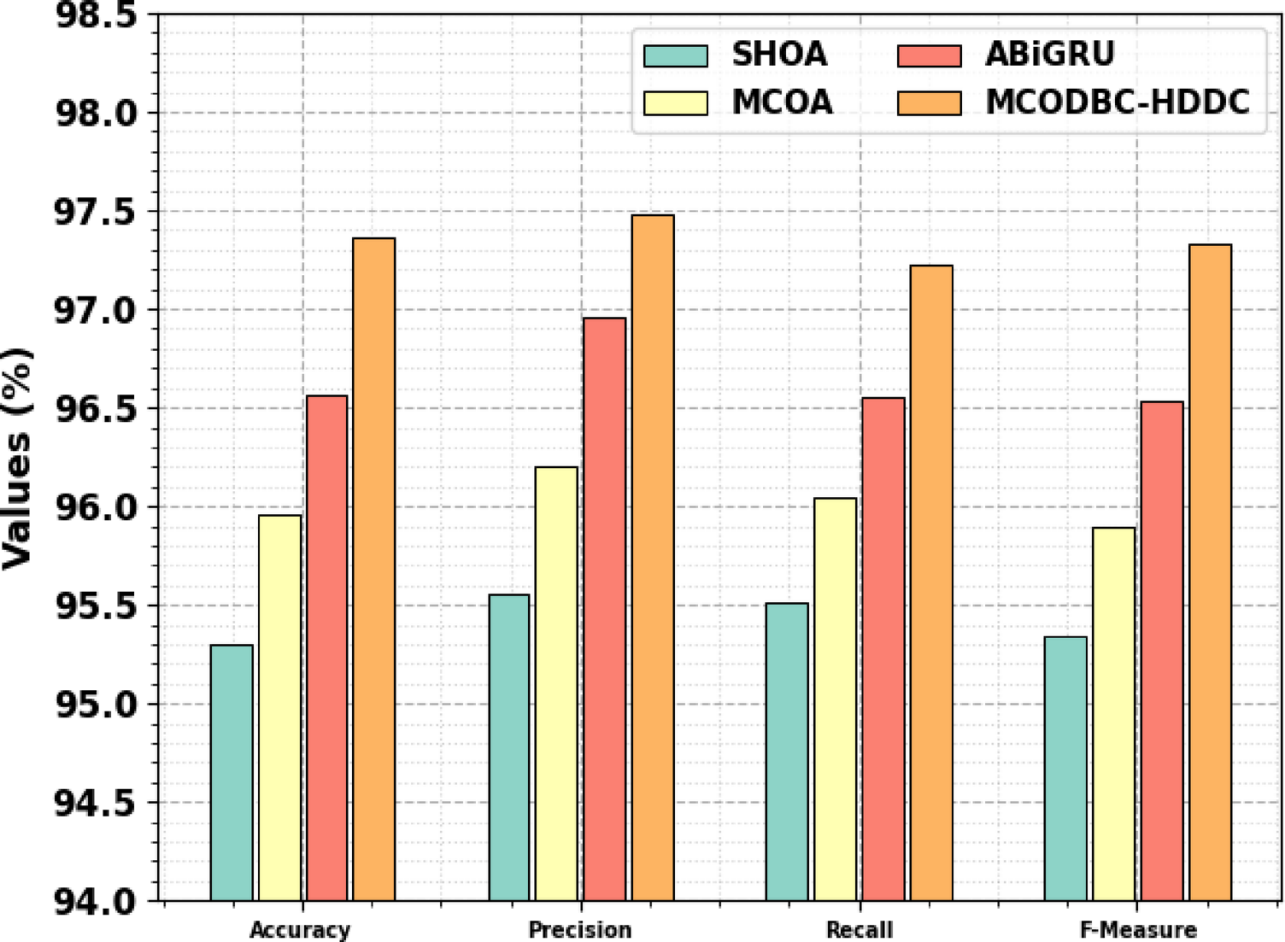
Result analysis of the ablation study of MCODBC-HDDC approach.

Table 6 and Fig. 11 specify the ablation study of the MCODBC-HDDC approach. The SHOA method demonstrated robust performance with an $acc{u}_{y}$ of 95.30, $pre{c}_{n}$ of 95.56, $rec{a}_{l}$ of 95.51, and an F-measure of 95.34. MCOA improved upon SHOA with an $acc{u}_{y}$ of 95.96, $pre{c}_{n}$ of 96.20, $rec{a}_{l}$ of 96.04, and a ${F}_{measure}$ of 95.90. ABiGRU improved these metrics, achieving an $acc{u}_{y}$ of 96.57, $pre{c}_{n}$ of 96.96, $rec{a}_{l}$ of 96.55, and a ${F}_{measure}$ of 96.53. MCODBC-HDDC method outperformed all other methods, attaining an $acc{u}_{y}$ of 97.36, $pre{c}_{n}$ of 97.48, $rec{a}_{l}$ of 97.22, and a ${F}_{measure}$ of 97.33. This study demonstrates the efficiency of these methods, specifically the MCODBC-HDDC model, in improving classification metrics for the given task.

**Table 6:** Result analysis of the ablation study of the MCODBC-HDDC approach;

Methodology	$Acc{u}_{y}$	$Pre{c}_{n}$	$Rec{a}_{l}$	${F}_{Measure}$
SHOA	95.30	95.56	95.51	95.34
MCOA	95.96	96.20	96.04	95.90
ABiGRU	96.57	96.96	96.55	96.53
MCODBC-HDDC	97.36	97.48	97.22	97.33

## Conclusions

In this manuscript, the MCODBC-HDDC method is proposed. The presented MCOBC-HDDC method provides an efficient and accurate disease diagnosis, utilizing a system based on DL techniques. To accomplish that, the MCODBC-HDDC method incorporates BC technology to ensure secure data sharing and management, providing a decentralized and tamper-proof environment for patient data. In the data preprocessing stage, the MCODBC-HDDC model employs Z-score normalization to standardize the data and improve performance. The SHOA is used to obtain the optimal subset of features. Besides, the ABiGRU method is implemented for disease detection and classification. Finally, the hyperparameter selection of the ABiGRU model is performed by utilizing the MCOA method. The experimental analysis of the MCODBC-HDDC approach is examined under the HD dataset. The performance validation of the MCODBC-HDDC approach portrayed a superior accuracy value of 97.36% over existing models. The limitations of the MCODBC-HDDC approach comprise the reliance on semi-synthetic datasets, which, while useful for controlled evaluation, may not fully capture the complexities of real-world clinical environments. The model’s performance may vary when applied to unstructured data sources, such as free-text medical notes or multi-modal records. Additionally, the model shows limitations in its adaptability in continuous monitoring scenarios. Future work may integrate real-time data streams and incorporate multi-source clinical data to improve model robustness. Expanding the model’s applicability across diverse healthcare systems and demographic groups is also a priority. Further research may explore the ethical and privacy considerations in deploying such intelligent systems in clinical practice. Validation using real-world, large-scale datasets will be pursued to assess practical effectiveness and scalability.

## Data Availability

The authors confirm that the data supporting the findings of this study are available within the article.
